# The treatment pattern of advanced HR-positive and HER2-negative breast cancer in central southern China: a hospital-based cross-sectional study

**DOI:** 10.1186/s12885-024-12665-0

**Published:** 2024-07-30

**Authors:** Zhe-Yu Hu, Binliang Liu, Ning Xie, Xiaohong Yang, Liping Liu, Huawu Xiao, Jing Li, Hui Wu, Jianxiang Gao, Jun Lu, Xuming Hu, Min Cao, Zhengrong Shui, Can Tian, Quchang Ouyang

**Affiliations:** 1 Medical Department of Breast Cancer, Hunan Cancer Hospital, No. 283, Tongzipo Road, Changsha, 410013 China; 2 Medical Department of Breast Cancer, The Affiliated Cancer Hospital of Xiangya School of Medicine, Central South University, Changsha, China

**Keywords:** Advanced breast cancer, HR-positive and HER2-negative, Diagnosis and treatment pattern, Investigation, Endocrine therapy

## Abstract

**Aims:**

This investigation aims to elucidate the treatment status of advanced HR+/HER2- breast cancer patients in Hunan Province of Central Southern China from November 2021 to December 2022.

**Methods:**

Data from 301 patients with advanced HR+/HER2- breast cancer were collected from the breast cancer investigation project in Hunan under the guidance of the Chinese Society of Clinical Oncolfogy (CSCO). The data included the clinical characteristics of patients and the status of first-line and second-line rescue treatment.

**Results:**

First-line chemotherapy and endocrine therapy for mBC accounted for 40% (121/301) and 60% (180/301) of treatments, respectively. AI (21%), AI plus CDK4/6 inhibitor (28%), and fulvestrant (24%) or fulvestrant plus CDK4/6 inhibitor (18%) were the most common first-line endocrine therapies. Taxane-based chemotherapy was the most common first-line chemotherapy (59%). Second-line chemotherapy and endocrine therapy for mBC accounted for 43% (72/166) and 57% (94/166) of treatments, respectively. Fulvestrant (23%) or fulvestrant plus CDK4/6 inhibitor (29%) were the most common second-line endocrine therapies. The prevalences of AI and AI plus CDK4/6 inhibitor decreased to 19% and 11%, respectively. T (taxane)-based chemotherapy was still the most common chemotherapy regimen (46%). Third-line chemotherapy was more prevalent than endocrine therapy (57% vs. 41%). T (taxane)-based chemotherapy was still the most common chemotherapy regimen (46%). Fulvestrant plus CDK4/6 inhibitor was the most common endocrine therapy (33%). AI, AI plus CDK4/6 inhibitor, and fulvestrant accounted for 21%, 12% and 18% of third-line endocrine therapies, respectively.

**Conclusions:**

Compared to chemotherapy, endocrine therapy was a more favorable choice for first-line and second-line treatment for HR+/HER2- advanced breast cancer patients in Hunan Province.

## Introduction

Both globally and in China, breast cancer has surpassed lung cancer to become the tumor with the highest number of new cases. In 2020, there were an estimated 2,261,419 new cases of breast cancer [1]. Among the current four subtypes of breast cancer, HR-positive and HER2-negative breast cancer is the most prevalent [2]. Compared with the United States, China has a higher proportion of advanced-stage breast cancer, and nearly two-thirds of breast cancer cases in China are diagnosed as advanced breast cancer (ABC) [3, 4].

Both international and Chinese guidelines recommend endocrine-based therapy as the standard treatment for HR-positive/HER2-negative metastatic breast cancer, except for in cases of visceral crisis [5–8]. The PALOMA series, MONARCH series and other studies have confirmed that the emergence of new drugs such as CDK4/6 inhibitors (palbociclib, abemaciclib, etc.) could improve the prognosis of HR-positive/HER2-negative mBC patients, regardless of endocrine sensitivity or endocrine resistance [9–15]. A meta-analysis of 140 studies (comprising 50,029 patients) suggests CDK4/6 inhibitors plus hormone therapies to be better than standard hormone therapy and supports the combination of hormone therapy plus targeted therapy (CDK4/6 inhibitor) as first- or second-line treatments [16]. Moreover, in a retrospective analysis of propensity score-matched patients receiving first-line chemotherapy (CT) versus first-line endocrine therapy (CT), no differences were observed between CT and ET in terms of overall survival (OS) or progression-free survival (PFS) [17].

To date, no descriptive study has examined the current treatment mode of HR-positive/HER2-negative mBC patients in Hunan Province, China. In clinical practice, there are still considerable differences in the treatment of HR-positive/HER2-negative mBC between China and Europe/the United States (US) [18]. A real-world study in China showed that [19] while the prevalence of initial endocrine treatment (ET) as the first-line treatment for HR-positive/HER2-negative mBC patients increased yearly from 1996 to 2018, the proportion of initial chemotherapy in Chinese patients was still higher than that in Europe or the US, reaching 64.7%. In 2018, palbociclib (the first CDK4/6 inhibitor) was approved for the treatment of HR-positive/HER2-negative mBC in China. Subsequently, several other new drugs were approved in China, including PIK3CA inhibitors, HDAC inhibitors, and other CDK4/6 inhibitors. Specifically, ribociclib (available in Europe) is currently not available in China, while dalpiciclib is only available in China. With the launch of new drugs, many changes have taken place in the treatment pattern of HR-positive/HER2-negative mBC patients. On the one hand, the prevalence of first-line endocrine therapy is expected to increase. On the other hand, after first-line standard ET combined with a CDK4/6 inhibitor, there is no currently approved PI3K inhibitor in China. It remains unclear how to arrange the treatment of second-line and later ET. In China, there are notable differences in treatment between different provinces, cities and rural areas.

Our research aims to explore the treatment mode of HR-positive/HER2-negative mBC patients in Hunan Province in 2021. We also examine the differences and causes of treatment decisions between Hunan Province and China as well as between Hunan Province and foreign countries. This study provides suggestions for improving the quality of diagnosis and treatment of HR-positive/HER2-negative mBC patients in China.

## Methods

### Study design and participants

**Fig. 1:**
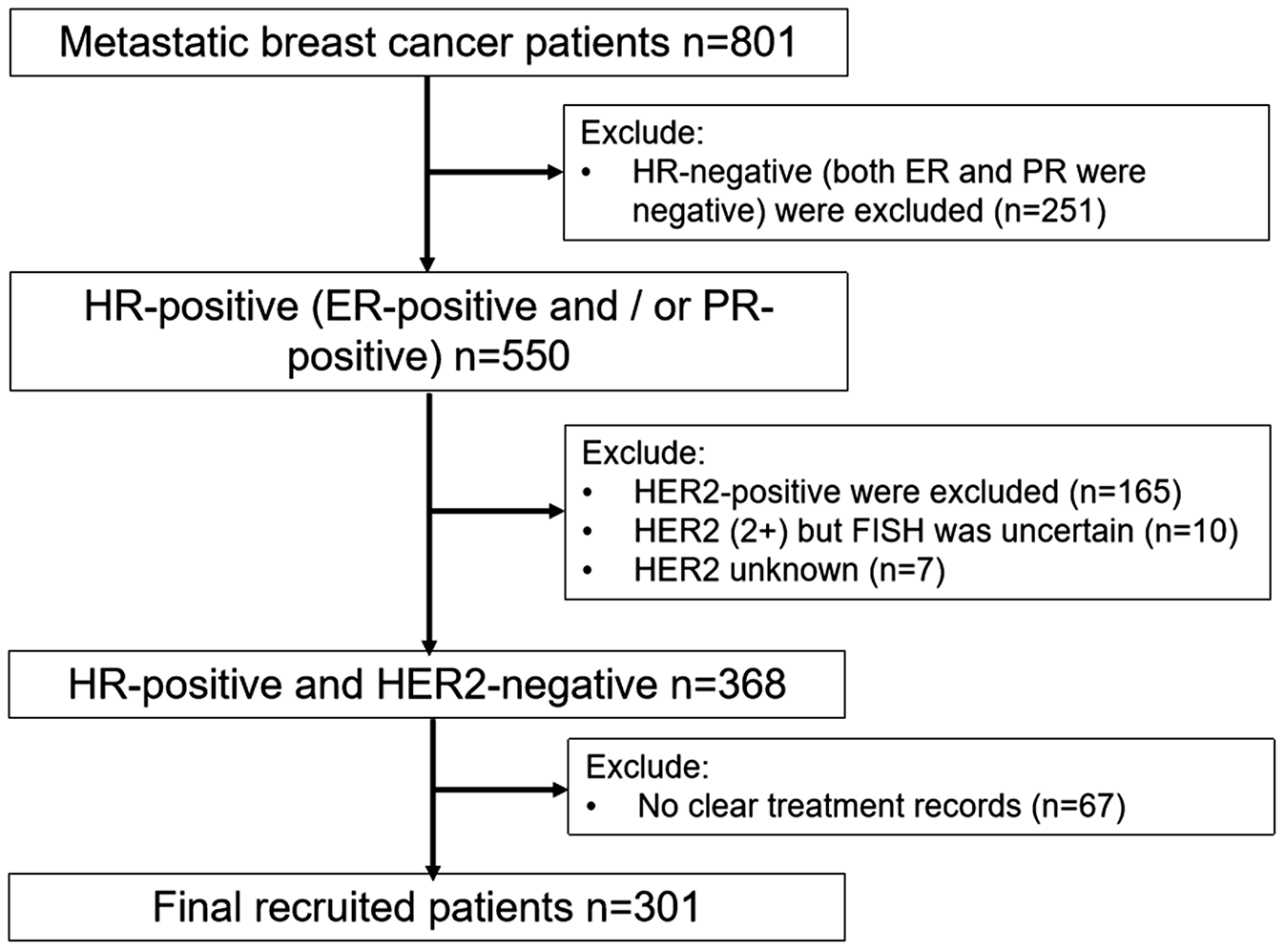
Patient enrollment flow

This was a hospital-based, noninterventional, retrospective study conducted from November 2021 to December 2022. Figure 1 shows the flow chart for patient enrollment. A total of 301 consecutive patients with HR-positive/HER2-negative mBC were enrolled in this study. The primary objective of this study was to describe the treatment pattern of HR-positive/HER2-negative mBC patients. The secondary objective was to describe the demographic and clinical features of HR-positive/HER2-negative mBC patients. ER- or PR-positivity was defined by the cutoff point of 1% of stained cells or a recording of positivity by physicians in medical records. Any ER- and/or PR-positive status was regarded as HR-positivity. HER2-negativity was defined as either an immunohistochemistry (IHC) score of 0, 1 + or 2 + and negative fluorescence in situ hybridization (FISH) or a recording of negative by physicians in medical records. First-line treatment was defined as initial therapy received for mBC up to first progression or therapy change. Chemotherapy (CT) alone was defined as the use of CT agents only. CT with maintenance therapy refers to the continuation of CT agents and/or endocrine agents after discontinuation of CT agents. Endocrine therapy (ET) was defined as the use of endocrine agents only.

**Fig. 2:**
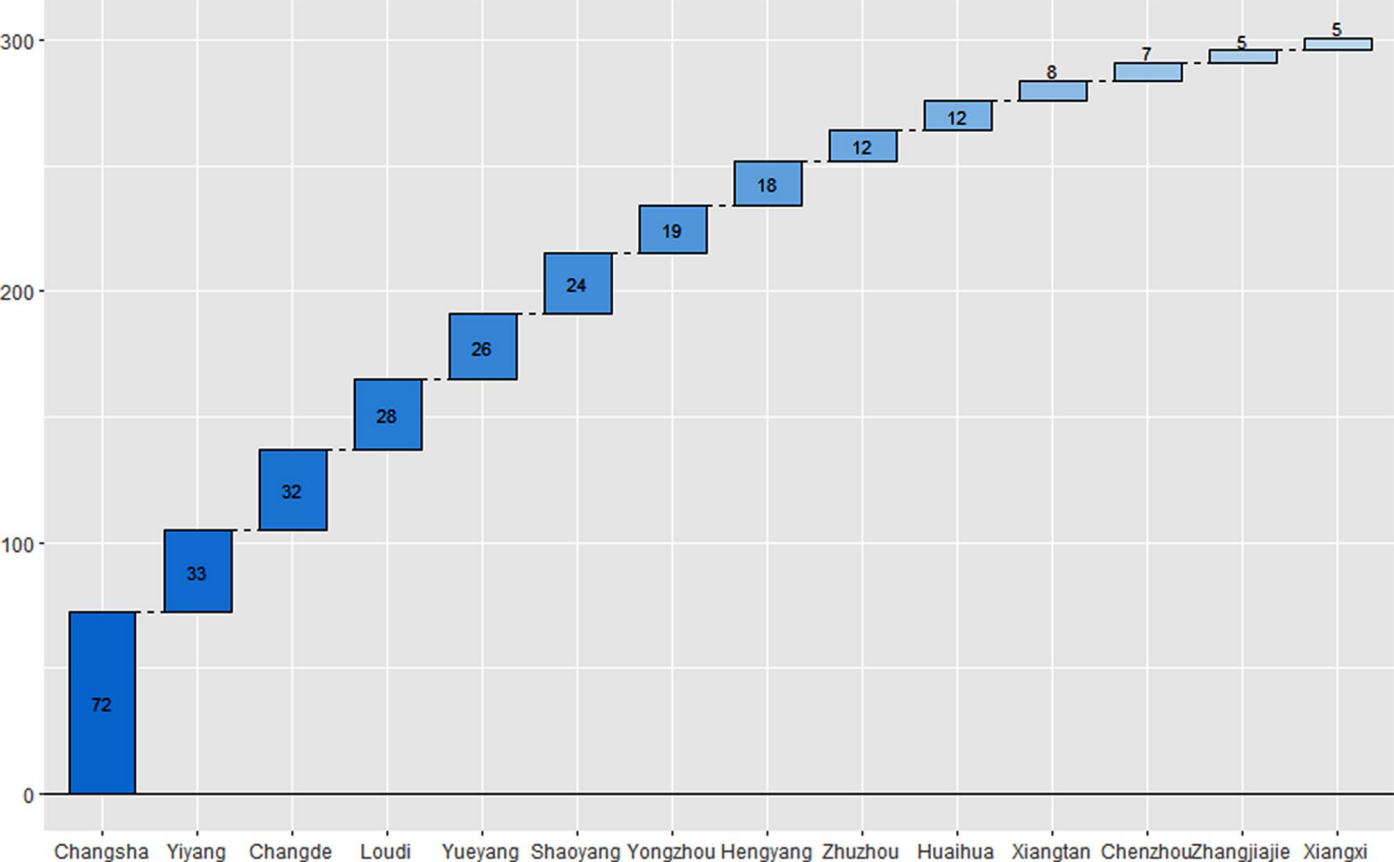
Area distribution of patients across hunan province

From November 2021 to December 2022, 301 cases of metastatic HR-positive and HER2-negative breast cancer patients collected by this project under the guidance of CSCO were selected as the survey subjects, all of whom were women from fourteen areas across Hunan Province (Fig. 2).

### General information and measurements

General information, including demographic, insurance and clinical information, was collected. The information included age at investigation, disease-free survival (DFS), number of metastatic sites (organs), estrogen receptor (ER) status, progesterone receptor (PR) status, treatment lines of mBC patients, endocrine therapy resistance, insurance type, and area (rural or urban). Descriptive analysis was performed to examine the use of first-, second-, and third-line chemotherapy or endocrine therapy. Treatments were first categorized into chemotherapy (CT) or endocrine therapy (ET). Then, CT was divided into treatment subgroups, such as T (taxane)-based, A (anthracycline)/E (epirubicin)-based, capecitabine (Cap), eribulin-based, G (gemcitabine)-based, N (navelbine)-based, and others. ET was also divided into treatment subgroups, including AI (aromatase inhibitor), AI plus CDK4/6 inhibitor, fulvestrant, fulvestrant plus CDK4/6 inhibitor, tamoxifen/torimifen, and other (HDAC inhibitor, PI3K inhibitor, etc.).

### Statistical description

Quantitative variables were reported as the mean (standard deviation) and median (interquartile range), and categorical variables were reported as counts (percentage). The distribution of patients among fourteen districts within Hunan Province was visualized using a waterfall plot, which was generated by R packages. The distributions of the first-, second- and third-line CT or ET for mBC patients were visualized using pie charts. The statistical analyses were primarily descriptive. Continuous variables were summarized mean (± standard deviation) and median (interquartile range). Categorical variables were reported as counts (percentage). Categorical variables were reported as counts (percentage). An analysis of variance was used to compare continuous variables with symmetrical distributions across subgroups. Chi-square tests and Fisher’s exact tests (*n* < 5) were used to compare categorical. Statistical analyses were conducted using SAS 9.4.

## Results

### Clinical characteristics

The clinical characteristics of all enrolled patients are listed in Table 1. The median age of mBC patients at the time of this investigation was 53 years (47–58 years), and the median disease-free survival (DFS) after surgery was 40 months (10–71 months). The numbers of patients who had one, two, three, four, or ≥ five metastatic sites (organs) were 100 (33.22%), 83 (27.57%), 62 (20.60%), 38 (12.62%), and 18 (5.98%), respectively. The ER-positivity and PR-positivity rates were high (> 10%) in 267 (88.70%) patients and 180 (59.80%), respectively. The number of patients with current first-, second-, third-, and ≥ fourth-line treatment for mBC was 134 (44.52%), 45 (14.95%), 46 (15.28%), and 76 (25.25%), respectively. Two hundred forty patients had endocrine therapy resistance. One hundred and ten (36.54%) patients had secondary endocrine therapy resistance. One hundred thirty (43.19%) patients had primary endocrine therapy resistance. Regarding the insurance type, approximately one-quarter of patients had employee medical insurance, with a fee coverage ranging from 70 to 90%. The majority of patients (134, 44.51%) used extracapital residence medical insurance, with fee coverage ranging from 30 to 50%. Forty-two (13.95%) patients were self-paid. The majority of patients (179, 59.47%) were rural residents. The distribution of all the enrolled patients across fourteen areas within Hunan Province is shown in Fig. 2.

**Table 1:** Patients’ characteristics

Variables	Level	Overall (*n*=301)
Age, years		52.68 ± 8.99, 53 (47, 58)
Disease free survival, months		50.3 ± 49.8, 40 (10, 71)
Number of Mets, n (%)	1	100 (33.22%)
	2	83 (27.57%)
	3	62 (20.60%)
	4	38 (12.62%)
	≥ 5	18 (5.98%)
ER, n (%)	Negative	22 (7.31%)
	≤ 10%	12 (3.99%)
	> 10%	267 (88.70%)
PR, n (%)	Negative	63 (20.93%)
	≤ 10%	58 (19.27%)
	> 10%	180 (59.80%)
Treatment lines for mBCs, n (%)	1	134 (44.52%)
	2	45 (14.95%)
	3	46 (15.28%)
	≥ 4	76 (25.25%)
Endocrine therapy resistance, n (%)	None	61 (20.27%)
	Secondary resistance	110 (36.54%)
	Primary resistance	130 (43.19%)
Insurance type, n (%)	Provincial employee medical insurance	10 (3.32%)
	Capital employee medical insurance	25 (8.31%)
	Extra-capital employee medical insurance	45 (14.95%)
	Capital residence medical insurance	38 (12.62%)
	Extra-capital residence medical insurance	134 (44.51%)
	Self-paid	42 (13.95%)
	Other	7 (2.32%)
Area, n (%)	Rural	179 (59.47%)
	Urban	122 (40.53%)

### Current treatment status

**Fig. 3:**
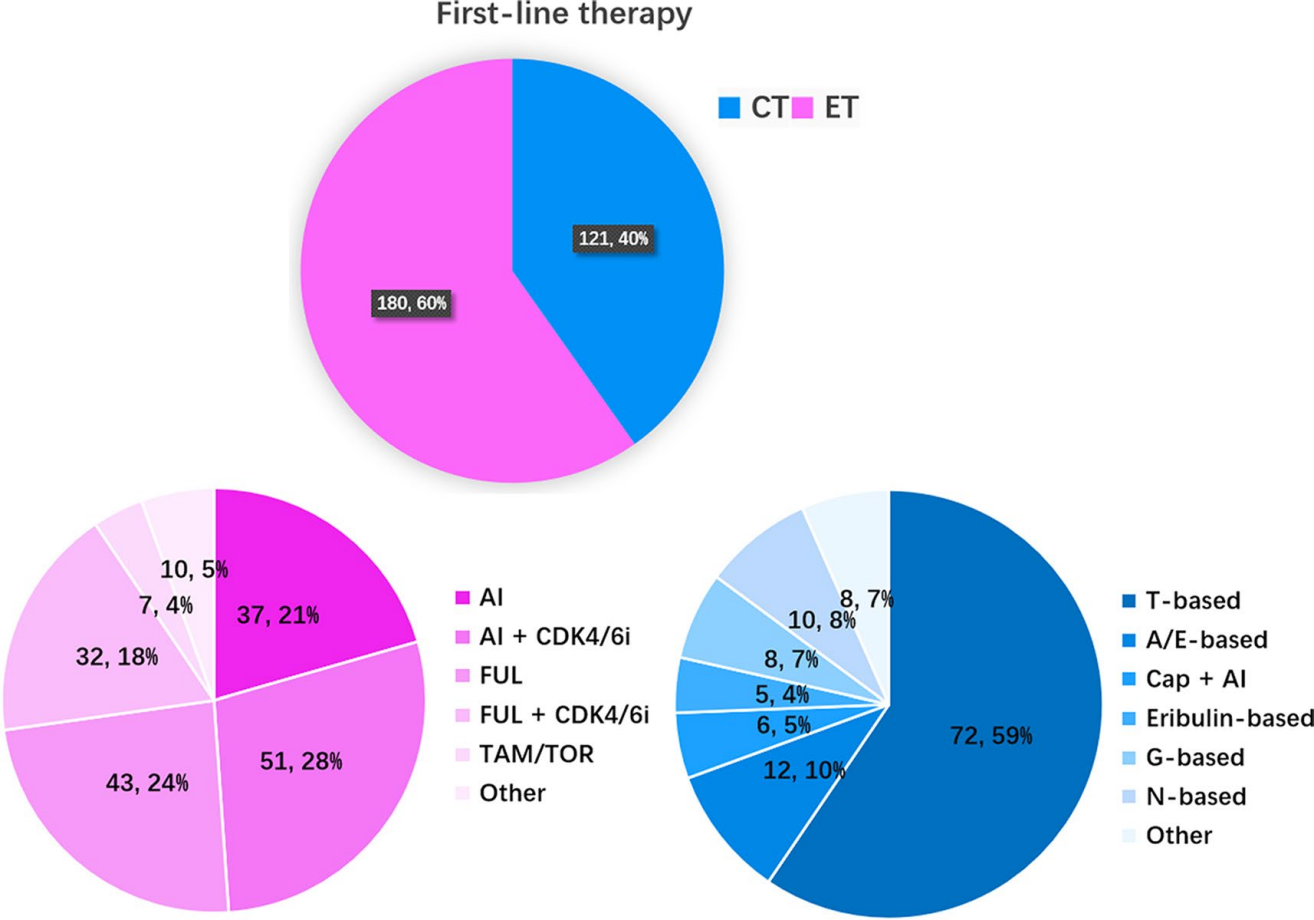
The CT and ET regimens as initial first-line treatment in patients with HR+/HER2– mBC. (**A**) Proportions of CT and ET as first-line treatment in different years (*n* = 1,877); (**B**) ET regimens as initial first-line treatment; (**C**) CT regimens as initial first-line treatment. T-based: single-agent taxane (T), TX, TP, GT, AT/TAC, and taxanes plus bevacizumab (T + Bev); N-based: single-agent vinorelbine, NX, NP; Cap: single-agent capecitabine; G-based: single-agent gemcitabine, GP, gemcitabine combined with capecitabine; A/E based: single-agent anthracycline (A/E), AC; Others: etoposide, 5-FU; AI: aromatase inhibitors; FUL: fulvestrant; ET + targeted drugs: endocrine therapy combined with targeted drugs, including CDK4/6 inhibitors, everolimus, and tucidinostat; TAM/TOR: tamoxifen, toremifene; MPA: megestrol, medroxyprogesterone

At enrollment, 135 (44.85%), 46 (15.28%), 45 (14.95%), and 75 (24.92%) patients received first-line, second-line, third-line and fourth- or above-line therapy for mBC, respectively. As shown in Fig. 3, first-line chemotherapy and endocrine therapy for mBC accounted for 40% (121/301) and 60% (180/301) of all treatments, respectively. AI (21%), AI plus CDK4/6 inhibitor (28%), and fulvestrant (24%) or fulvestrant plus CDK4/6 inhibitor (18%) were the most common first-line endocrine therapies. Tamoxifen or torimifen accounted for 4% of first-line endocrine therapies. Other first-line endocrine therapies included AI or fulvestrant plus an HDAC inhibitor or PI3K inhibitor (5%). For first-line chemotherapy, T (taxane)-based chemotherapy was the main first-line chemotherapy (59%). Patients receiving A/E-based, capecitabine plus AI, eribulin-based, G-based, and N-based treatments accounted for 10%, 5%, 4%, 7%, and 8%, respectively. Other first-line chemotherapies included A/E + capecitabine, capecitabine alone, GT + AI, etc.

**Fig. 4:**
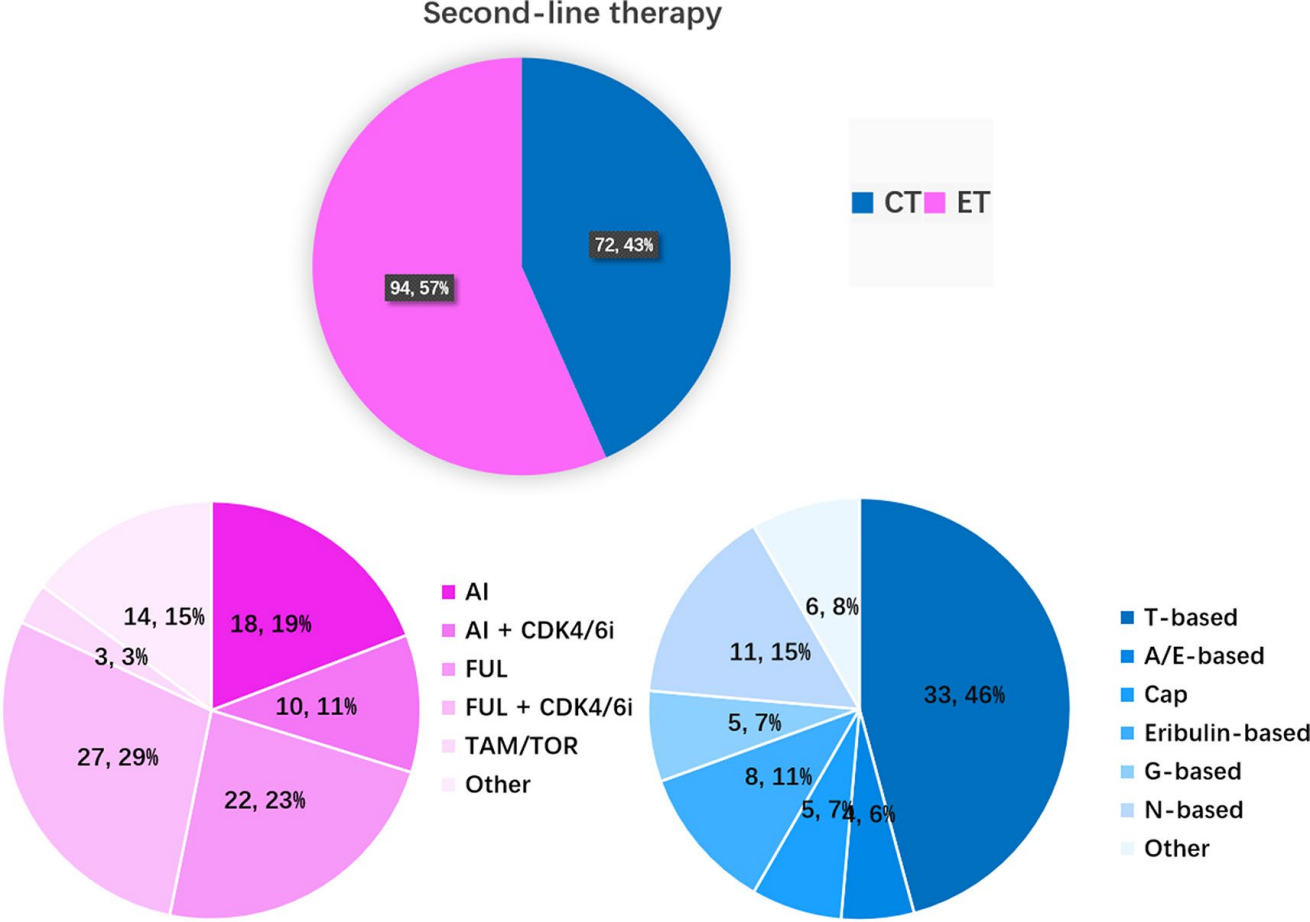
The CT and ET regimens as second-line treatments in patients with HR+/HER2– mBC. (**A**) Proportions of CT and ET as first-line treatment in different years (*n* = 1,877); (**B**) ET regimens as initial first-line treatment; (**C**) CT regimens as initial first-line treatment. T-based: single-agent taxane (T), TX, TP, GT, AT/TAC, and taxanes plus bevacizumab (T + Bev); N-based: single-agent vinorelbine, NX, NP; Cap: single-agent capecitabine; G-based: single-agent gemcitabine, GP, gemcitabine combined with capecitabine; A/E based: single-agent anthracycline (A/E), AC; Others: etoposide, 5-FU; AI: aromatase inhibitors; FUL: fulvestrant; ET + targeted drugs: endocrine therapy combined with targeted drugs, including CDK4/6 inhibitors, everolimus, and tucidinostat; TAM/TOR: tamoxifen, toremifene; MPA: megestrol, medroxyprogesterone

Second-line chemotherapy and endocrine therapy for mBC accounted for 43% (72/166) and 57% (94/166) of treatments, respectively (Fig. 4). Fulvestrant (23%) or fulvestrant plus CDK4/6 inhibitor (29%) were the most common second-line endocrine therapies. The prevalences of AI or AI plus CDK4/6 inhibitor treatment decreased to 19% and 11%, respectively. T (taxane)-based chemotherapy was still the most common chemotherapy regimen (46%). A/E-based, capecitabine, eribulin-based, G-based, and N-based treatments accounted for 6%, 7%, 11%, 7%, and 15% of second-line endocrine therapies, respectively. Other second-line chemotherapies included capecitabine plus fulvestrant, TROP2 antibody‒drug conjugates (ADCs) [20–22] etc.

**Fig. 5:**
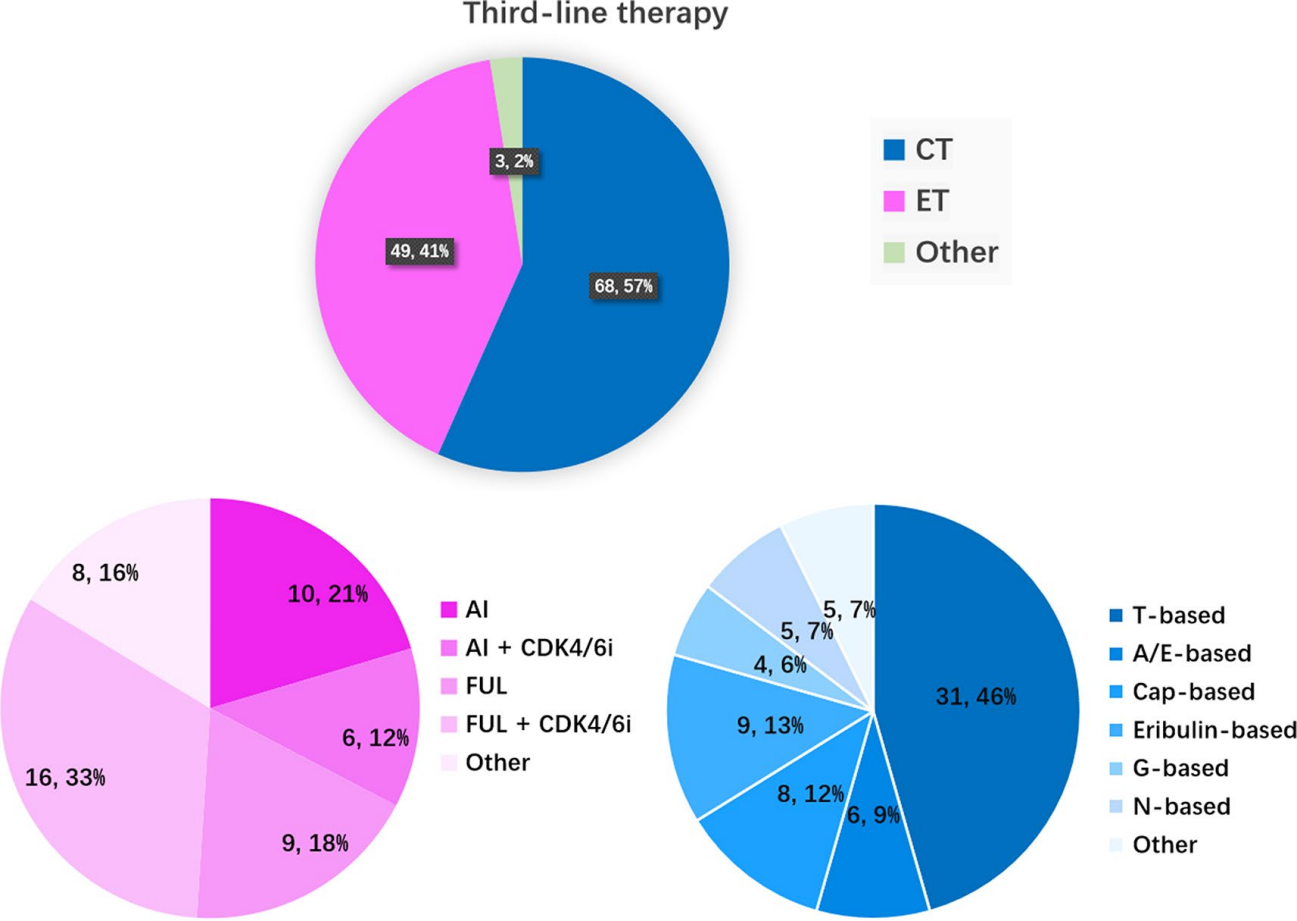
The CT and ET regimens as third-line treatment in patients with HR+/HER2– mBC. (**A**) Proportions of CT and ET as first-line treatment in different years (*n* = 1,877); (**B**) ET regimens as initial first-line treatment; (**C**) CT regimens as initial first-line treatment. T-based: single-agent taxane (T), TX, TP, GT, AT/TAC, and taxanes plus bevacizumab (T + Bev); N-based: single-agent vinorelbine, NX, NP; Cap: single-agent capecitabine; G-based: single-agent gemcitabine; GP, gemcitabine combined with capecitabine; A/E based: single-agent anthracycline (A/E), AC; Others: etoposide, 5-FU; AI: aromatase inhibitors; FUL: fulvestrant; ET + targeted drugs: endocrine therapy combined with targeted drugs, including CDK4/6 inhibitors, everolimus, and tucidinostat; TAM/TOR: tamoxifen, toremifene; MPA: megestrol, medroxyprogesterone

Third-line chemotherapy was more common than endocrine therapy (57% vs. 41%). T (taxane)-based chemotherapy was still the main chemotherapy regimen (46%). A/E-based, capecitabine-based, eribulin-based, G-based, and N-based treatments accounted for 9%, 12%, 13%, 6%, and 7% of third-line chemotherapies, respectively. Other third-line chemotherapies included capecitabine plus fulvestrant, TROP2 ADCs, etc. Fulvestrant plus CDK4/6 inhibitor was the main endocrine therapy (33%). AI, AI plus CDK4/6 inhibitor, and fulvestrant accounted for 21%, 12% and 18% of third-line endocrine therapies, respectively. The prevalence of other third-line endocrine therapies (AI or fulvestrant plus HDAC inhibitor or PI3K inhibitor) increased to 16% (Fig. 5).

### Endocrine therapy-related clinical trials

A high number of patients who received endocrine therapy reported previously participating in clinical trials. As shown in Table 2, among 180 patients who received first-line endocrine therapy, 29 (16%) patients participated in clinical trials. Among these 29 patients, 24 (82.8%) participated in CDK4/6i-involved clinical trials. Among 94 patients who received second-line endocrine therapy, 6 (6.4%) patients participated in clinical trials, and all these patients participated in CDK4/6i-involved clinical trials. Among 49 patients who received third-line endocrine therapy, 5 (10%) patients participated in clinical trials, and two of them participated in CDK4/6i-involved clinical trials. Other agents in clinical trials included PI3K inhibitors and HDAC inhibitors.

**Table 2:** Endocrine therapy-related clinical trials in HR+/HER2- advanced breast cancer patients

Endocrine therapy	Clinical trials	CDK4/6 inhibitor-involved clinical trials
First-line	29	24
Second-line	6	6
Third-line	5	2

### AI agents and CDK4/6 inhibitors

Regarding the treatment agents, Table 3 shows that among the 180 patients who received first-line endocrine therapy (Figs. 3), 81 (49%) received AI treatment. Among these 89 patients, 31 received letrozole, 38 received anastrozole, and 27 received exemestane. Among the 94 patients who received second-line endocrine therapy, 28 (30%) received AI treatment (Fig. 4). Among these 28 patients, 13 received letrozole, 10 received anastrozole, and 8 received exemestane. Among the 49 patients who received third-line endocrine therapy, 16 (33%) received AI treatment (Fig. 5). Among these 16 patients, 1 received letrozole, 9 received anastrozole, and 9 received exemestane.

**Table 3:** The usage of AI agents in HR+/HER2- advanced breast cancer patients

Endocrine therapy	AI agents	Numbers
First-line	Letrozole	31
	Anatrozole	38
	Exemestane	27
Second-line	Letrozole	13
	Anatrozole	10
	Exemestane	8
Third-line	Letrozole	1
	Anatrozole	9
	Exemestane	9

Regarding CDK4/6 inhibitors, Table 4 shows that among the 180 patients who received first-line endocrine therapy (Figs. 3), 83 (46%) received CDK4/6 inhibitor treatment. Among these 83 patients, 34 received abemaciclib, 25 received palbociclib, and 24 received CDK4/6i-involved clinical trials. Among the 94 patients who received second-line endocrine therapy (Figs. 4), 36 (38%) received CDK4/6 inhibitor treatment. Among these 36 patients, 10 received abemaciclib, 18 received palbociclib, 2 received dalpiciclib, and 6 received CDK4/6i during clinical trials. Among the 49 patients who received second-line endocrine therapy (Figs. 5), 22 (45%) received CDK4/6 inhibitor treatment. Among these 22 patients, 10 received abemaciclib, 11 received palbociclib, and 2 received CDK4/6i during involved clinical trials. Notably, ribociclib is currently not available (like in Europe), while dalpiciclib is only available in China. Regional differences leading to medication differences are also noteworthy.

**Table 4:** The usage of CDK4/6 inhibitors in HR+/HER2- advanced breast cancer patients

Endocrine therapy	CDK4/6 inhibitors	Numbers
First-line	Abemaciclib	34
	Palbociclib	22
	Dalpiciclib	0
Second-line	Abemaciclib	10
	Palbociclib	18
	Dalpiciclib	2
Third-line	Abemaciclib	10
	Palbociclib	11
	Dalpiciclib	0

## Discussion

The prognosis of mBCs has been verified to be better with CDK4/6 plus AI or fulvestrant, especially the early-line use, in Europe [23, 24], the US [25] and East Assia [26–28]. However, adding CDK4/6 inhibitor with a high price to a regimen of endocrine therapy was not cost-effective in 2019–2022 in both the US and China [29, 30]. Currently in 2024, since the price of CDK4/6 has decreased to about 10-20% of the original price, it becomes a prevalent option for HR+/HER2- MBCs in China. In addition, a Canadian real-world study suggests CDK4/6 plus with ET to be well tolerated with a better prognosis [31]. People with mBCs face many challenges and disparities in obtaining optimal cancer care in both Europe [32] and China. There is clearly an unmet medical need for access to innovative treatment including CDK4/6 for women with mBCs in China. Compared with some countries where these treatments are 100% reimbursed, the majority of patients are partly reimbursed and even some are self-paid in China (Table 1).

Compared to patients across Europe, approximately 40% of the HR-positive patients in the current cohort received CDK4/6 inhibitor treatment [32]. In Hunan Province, approximately 30% of HR-positive/HER2-negative patients received first-line CDK4/6 inhibitor treatment, approximately 23% of patients received second-line CDK4/6 inhibitor treatment, and approximately 18% of patients received third-line CDK4/6 inhibitor treatment. In total, the majority of patients in Hunan Province have received CDK4/6 inhibitor treatment. A real-world study across China [19] indicated that the proportion of patients who received first-line endocrine therapy increased from 25.4% in 1996–2005 to 44.6% in 2016–2018. In Hunan Province, the proportion of patients receiving first-line endocrine therapy exceeded that of first-line chemotherapy in 2021–2022 (60% vs. 40%, Fig. 3). The proportion of patients receiving second-line endocrine therapy also exceeded that of second-line chemotherapy (57% vs. 43%, Fig. 4). After two lines of endocrine therapy, the proportion of patients receiving chemotherapy exceeded endocrine therapy in the third-line treatment (57% vs. 41%, Fig. 5). Other drugs, such as TROP2 ADCs [21], were also used in third-line therapy.

However, in this study, although the recruited patients were distributed across all fourteen areas of Hunan Province (Fig. 2), all of them were treated in Hunan Cancer Hospital. Therefore, the findings of this study could not represent all the treatment conditions around Hunan Province; instead, this study could only reflect the treatment level in Hunan Cancer Hospital. This real-world study did not collect medical data from other hospitals, so the treatment strategies in other areas of Hunan Province were not analyzed herein.

Even though the treatment strategy could not represent the entirety of Hunan Province, the patients’ condition is representative of the overall breast cancer patients across Hunan Province. As shown in Table 1, the median age of mBC patients at the time of this investigation was 53 years (47–58 years), and the median disease-free survival (DFS) after surgery was 40 months (10–71 months). A total of 43.19% of patients had primary endocrine therapy resistance, and 36.54% of patients had secondary endocrine therapy resistance. Only approximately one-quarter of patients had employee medical insurance, with a fee coverage ranging from 70 to 90%. The majority of patients (44.51%) used extra-capital residence medical insurance, with a fee coverage ranging from 30 to 50%. A total of 13.95% of patients were self-paid. In addition, since CDK4/6 inhibitors were not covered by Chinese insurance, the fee was much higher than that for European patients. Even so, the proportion of patients in Hunan Cancer Hospital using CDK4/6 inhibitors was still not low. Many patients become poor due to cancer treatment. Therefore, we suggest that the government expand medical insurance coverage for effective drugs and treatment fees.

In this study, we found that the proportion of patients receiving first- or second-line chemotherapy was close to 40%, which may be related to this group of patients receiving front-line treatment in grassroots hospitals. Therefore, we need to further enhance knowledge regarding standard treatment philosophies in grassroots hospitals. The clinical practice and guidelines of basic hospitals for HR+/HER2- advanced breast cancer is still very different. For patients receiving first-line endocrine therapy, only 46% of them have used CDK4/6 inhibitors combined with endocrine therapy, which obviously leaves much room for improvement. To increase the proportion of combined therapy, it is important for the current treatment model to improve the diagnosis and treatment quality of HR+/HER2- advanced breast cancer. Therefore, enhancing knowledge regarding standard treatment philosophies is our consistent recommendation.

Another reason for the low proportion of first-line CDK4/6 inhibitor treatment in Hunan, a central province of China, may be the high price of drugs or the lack of access to CDK4/6 inhibitors in grassroots hospitals. Therefore, the government and manufacturers should increase the accessibility of drugs.

We also need to strengthen the cooperation between superior and subordinate hospitals. Currently, a breast cancer specialty alliance has been established in Hunan Province, covering many multilevel hospitals at the provincial, municipal and county levels (if the number can be added). However, this program is far from sufficient for the whole Hunan Province. We should continue to increase the scale of cooperation between superior and subordinate hospitals, establish an effective cooperation system and model, promote the necessary referral treatment of patients, and obtain timely and standard treatment.

Multidisciplinary cooperation is important. Breast cancer patients should pay more attention to the whole process management of patients, so the improvement of HR+/HER2- advanced breast cancer diagnosis and treatment quality must be based on multidisciplinary cooperation, regular multidisciplinary cooperation, a more comprehensive understanding of patient characteristics, and enabling patients to receive more standard and personalized treatment.

Regardless of the kind of treatment method, the ultimate goal of CDK4/6 inhibitors treatment should be to benefit patients [33–35]. Attention should be devoted to patient education, and patient education activities should be regularly carried out so that patients can better understand the most beneficial treatment method [36]. In addition, compared with other subtypes, the prognosis of HR+/HER2- advanced breast cancer patients are relatively better. Therefore, for these patients, the efficacy of treatment should be emphasized, and the quality of life should be considered when evaluating therapeutic options.

This study has some limitations. First, since this is a single-center/ hospital-based study, the results might not be able to representative for the whole country. Second, retrospective data might cause some bias. Concerning the low insurance coverage for CDK4/6 inhibitor and the high self-paid rate, Hunan Provincial government has increased the reimbursement rate to 60-95% (according to different hospital grades, patients’ economic situations and insurance type) for CDK4/6 inhibitors (except Ribociclib) from 2023. Moreover, the price of CDK4/6 decreased significantly from about 30,000 RMB in 2019 to about 4,000 RMB in 2023. With the guideline recommendation and effective policy, we hope that the prevalent rate will reach 100%.

## Data Availability

Data will be made available upon reasonable request (contact Zhe-Yu Hu, email: huzheyu@hnca.org.cn).

## Abbreviations

HER2: Human epidermal growth factor receptor 2
HR: Hormone receptor
ER: Estrogen receptor
mBC: Metastatic breast cancer
AI: Aromatase inhibitor
PFS: Progression-free survival
OS: Overall survival
TKI: Tyrosine kinase inhibitor
OFS: Ovarian function suppression
RECIST: The Response Evaluation Criteria in Solid Tumors
ECOG: Eastern Cooperative Oncology Group
CNS: Central nervous system
AEs: Adverse events
NCI-CTCAE: The National Cancer Institute Common Terminology Criteria for Adverse Events
CBR: Clinical benefit rate
CR: Complete response
PR: Partial response
SD: Stable disease
ORR: Objective response rate
CI: Confidence interval
